# Triple Arthrodesis with Concomitant Tendo-Achilles Lengthening in Stage III Adult Acquired Flatfoot – A Retrospective Study of Prospectively Collected Clinical and Radiological Outcomes at Two Years

**DOI:** 10.5704/MOJ.2511.009

**Published:** 2025-11

**Authors:** JY Lee, SJ Wong, IS Rikhraj

**Affiliations:** 1 Department of Orthopaedic Surgery, Singapore General Hospital, Singapore; 2 Department of Orthopaedic Surgery, Sengkang General Hospital, Singapore

**Keywords:** adult acquired flat foot deformity, triple arthrodesis, tendo-achilles lengthening, AOFAS score, calcaneal pitch

## Abstract

**Introduction:**

Triple Arthrodesis is the preferred surgical treatment of stage III Adult Acquired Flatfoot Deformity. However, studies have reported subsequent midfoot degenerative arthrosis, with medial column instability.

**Material and Methods:**

This is a retrospective study using prospectively collected registry data from a single tertiary institution. Between 2007 to 2014, all patients who had undergone Triple Arthrodesis with concomitant percutaneous Tendo-Achilles lengthening were included. Patients with previous foot surgery, revision surgeries, and concomitant surgical procedures were excluded. We aim to report on our two years outcomes using the hindfoot and midfoot AOFAS and VAS scores, as well as radiographic parameters such as first-metatarsal declination angle, medial cuneiform calcaneal angle, navicular height, medial cuneiform – first metatarsal cuneiform angle, calcaneal pitch and medial longitudinal arch.

**Results:**

There were 22 feet from 21 patients that met the criteria of having undergone Triple Arthrodesis with concomitant percutaneous Tendo-Achilles lengthening. Hindfoot and midfoot American Orthopaedic Foot and Ankle Society (AOFAS) scores improved by an average of 34.6 and 36.7 points at 24-months post-surgery (p<0.001). Between six months to two years post-operatively, there was evidence of mid-foot sagging as shown with an increase in Calcaneal-First metatarsal angle by a mean of 5.1° (p=0.001). Additionally, a decrease in the Calcaneal Pitch Angle by 2.9° (p=0.003) and Cuneiform-Calcaneal angle by 5.5° (p=0.000). Navicular Height also decreased significantly by 2.8mm (p=0.001) over this period. A total of 18 (85.6%) reported overall satisfaction being met at the time of a phone survey, at an average of 8.3 years (range 5.8 to 12.4) post-surgery. One patient required an ankle replacement, and another had removal of hardware done.

**Conclusion:**

Triple arthrodesis with concomitant Tendo-Achilles lengthening results in significant improvement in AOFAS mid-foot and hindfoot scores at two years. At two years mark, there was features of mid-foot sagging postoperatively, despite continued improvement in AOFAS scores. Eighteen of the 21 patients were satisfied with the outcomes, on a phone survey, at an average of 8.3 years post-surgery.

## Introduction

Adult Acquired Flatfoot Deformity (AAFD) is a biomechanical complex deformity comprising of forefoot abduction, midfoot arch collapse, and hindfoot valgus^1^. The Triple Arthrodesis (TAO) first described by Ryerson^2^ has become a highly successful surgery correcting the deformity, relieving both mechanical and arthritic pain in grade III AAFD on the modified Johnson and Strom classification scale^3-6^. The goals of a triple arthrodesis in AAFD are pain relief, correction of the flat foot deformity with a stable realignment, and the creation of a balanced plantigrade foot for ambulation^7^. However, long-term studies show that patients post triple arthrodesis subsequently develop adjacent midfoot degenerative arthrosis, typically at the naviculocuneiform and metatarsocuneiform joints^8,9^. Medial column instability has also been reported following Triple Arthrodesis by Meary who reported mid-term arch collapse beyond pre-operative angles when assessed three years postoperatively^10^. In his series, there was significant naviculocuneiform instability and loss of calcaneal pitch from baseline radiographic measurements.

The contracted Achilles Tendon (TA) is involved in the pathogenesis of AAFD and as an effect of end stage arch collapse^11-15^. It needs to be addressed as part of comprehensive AAFD deformity correction^16,17^. Catanzariti *et al* recommends either concurrent Achilles Tendon Lengthening (TAL) or gastrocnemius recession in all patients undergoing triple arthrodesis to restore and maintain a plantigrade foot^18^. Despite the evidence of TA contracture in AAFD and the importance of addressing it during surgery, there is a paucity of literature examining TAL in hindfoot fusion procedures for AAFD^19^.

We aim to report on our two years patient report outcomes using the AOFAS hindfoot and midfoot scores, hindfoot and midfoot VAS scores, as well as radiographic measurements such as Calcaneal Pitch Angle, Medial Longitudinal Arch, First-metatarsal Declination Angle, Navicular Height, Medial Cuneiform Calcaneal Angle and Medial Cuneiform – First Metatarsal Cuneiform Angle.

## Materials and Methods

We performed a retrospective analysis of prospectively collected data, involving patients who had undergone TAO with percutaneous TAL. Institutional Review Board approval was obtained under local ethics committee (Singhealth CIRB 2017/2832). Between 2007 to 2014, all patients who failed minimum 6 months of conservative treatment of stage III AAFD on the modified Johnson and Strom classification and had undergone TAO with percutaneous TAL were included in the study. The cases are a single surgeon series, the senior author of this paper. All the 21 patients (22 feet) had an Achilles Tendon contracture on clinical examination with the affected foot being in equines of 5° or more and the Silfverskiöld test^20^ used to exclude a gastrocnemius contracture.

We excluded any patient who had (1) prior subtalar arthrodesis (2) fractures of the foot leading to secondary pes planus deformity (3) concomitant procedures performed at the same sitting other than Achilles tendon lengthening (for example medial column or lateral column surgery) or (4) minimally invasive TAO.

The surgery was performed by the senior author at our institution. Patients were positioned supine with a thigh tourniquet. Percutaneous Tendo-Achilles lengthening was first carried out using a modified two-step cut technique according to the surgical methods described by Hansen^21^. The Tendo-Achilles was placed in tension by dorsiflexing the foot. Surgical incisions were made at the postero-lateral border of the Tendo-Achilles about 1 – 1.5cm above insertion of the Tendo-Achilles and a second incision was made 1.5 – 2cm above the first incision at the midline of the Tendo-Achilles. On immediate completion of the lengthening procedure, there would be a sudden give experienced and a gap palpable at the skin interval been the two incisions. A standard triple arthrodesis was then performed through two incisions. After cartilage excision at all six joint surfaces, the joints were packed with cancellous bone graft harvested from the ipsilateral upper tibial metaphysis through a cortical window made at Gerdy’s tubercle. The foot was checked clinically and radiologically on the table, to ensure the transverse and longitudinal arches were restored after manipulation of the foot and temporary fixation with K-wires. The final fixation of the subtalar joint was with a 6.5mm partially threaded cannulated screw [Synthes-Depuy, Warsaw Indiana], while the talonavicular and calcaneocuboid joints were fixed with x-plates [Synthes-Depuy, Warsaw Indiana]. Surgical wounds were closed in layers with non-absorbable sutures used for skin closure. A U-slab stirrup plaster was applied immediately postoperatively and this was changed to a below-knee full plaster, with the foot in plantigrade, on the first postoperative day. Patients were discharged on the second postoperative day on a pair of crutches and instructed to non-weight bear on the operated leg. Patients were reviewed at two to three weeks post-surgery and sutures were removed once the surgical incisions had healed. Patients were then transitioned into a long Aircast [ANKL Inc, DJO©, CA, USA] orthopaedic boot and advised weight-bearing as tolerated, with increments in weight-bearing as the patient became more comfortable with walking. Gentle ankle range of motion exercises were commenced at four weeks postoperatively. All patients were seen at eight weeks post-surgery with radiographs. Radiographs revealed that fusion had occurred and clinically the Tendo-Achilles was checked to have healed. Patients then had the Aircast boot removed and were placed on an active walking and limb-strengthening program.

**Fig. 1:**
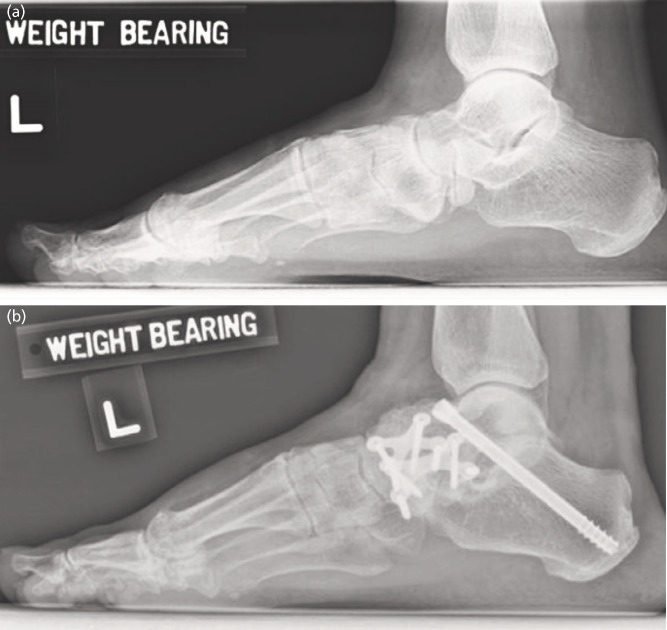
Weight bearing radiographs (a) pre-surgery and (b) six months post-surgery.

Weight-bearing dorsoplantar and lateral radiographs of the foot were taken (1) pre-surgery, (2) eight weeks and six months post-surgery and (3) two years post-surgery (Fig. 1). Medial Cuneiform-Calcaneal Angle (CCA) and Medial Cuneiform-1st Metatarsal Angle (MCA) indicated the stability of the midfoot joints in the sagittal plane^10^. The grades of osteoarthritis at the naviculocuneiform and medial cuneiform-1st metatarsal joints were determined with the Kellgren and Lawrence score^22^. One grade increase on the Kellgren and Lawrence score over time was considered an aggravation in osteoarthritis when comparing two years to six months post-operative radiographs.

Pre-operative and post-operative scores, at six months and two years, were collected prospectively by trained physiotherapists. Clinical outcomes assessed included midfoot and hindfoot pain and American Orthopaedic Foot and Ankle Society (AOFAS) midfoot and hindfoot scores. Pain related specifically to the midfoot and hindfoot was measured using a visual analogue scale (VAS)^23^. AOFAS scores for midfoot and hindfoot have specific outcome measures with 40 points assigned to pain, 45 points assigned to function, and 15 points assigned to alignment, with higher scores indicating better function, alignment and less pain^24^. Patient satisfaction was assessed at two years postoperatively using questions 53 and 48 adapted from the North American Spine Society (NASS) Questionnaire. Scores were then converted into a binary scale of whether overall satisfaction was met.

A phone survey was conducted to enquire about their VAS, physical function and satisfaction. Physical function was assessed based on what walking aids they required and how ambulant they were. Satisfaction score was based on the same North American Spine Society (NASS) Questionnaire as detailed above. We also enquired whether they had further surgery done to the same foot. At the time of phone survey, an average of 8.3 (range 5.8 to 12.4) years had passed since the index surgery.

All statistical analysis was performed with SPSS Statistics for Windows, Version 26.0. Armonk, NY: IBM Corp Released 2019. Equality of variances was calculated to determine the T-test statistic. Pearson’s coefficient was used to correlate clinical, radiological and satisfaction scores. P-values less than 0.05 were considered statistically significant and 95% Confidence Intervals (CI) were calculated.

## Results

There were 22 feet from 21 patients that underwent TAO with TAL. Sixteen (85.7%) were female and 5 (23.8) were male. The average age of the patients at the time of operation was 62.1 (range 50.6 to 78.3) and their average BMI was 30.2 (Range 21.1 to 43.5). At the time of the phone survey, patients were on average 70.2 years of age (range 57.2 to 87.5). Two (9.1%) patients who had undergone further surgeries to the index foot.

Pre-operative averages of NH, Pitch, Longitudinal Arch and M1 were out of the reference ranges for normal feet and reflected planar deformation. After Triple Arthrodesis with TAL, NH, Pitch, Arch and M1 were corrected significantly (p<0.05) at six months post-operatively when compared to pre-surgery (Table I). The average angles for all measurements at six months post-surgery converged towards predefined angles of a “normal” foot based on previously published results^25,26^. Pitch, Longitudinal Arch and M1 six months post-surgery were corrected to within one standard deviation of previously published averages^25^.

**Table I:** Immediate post-surgery and two years post-surgery measurements compared to pre-operative measurements, paired T-test. All measurements of angles are presented in degrees (°), and Navicular height presented in millimetres (mm).

	Pre-surgery	Six months post-surgery	95% CI of the difference		p-value	Two years post-surgery	95% CI of the difference		p-value
			Lower	Upper			Lower	Upper	
Pitch °	7.8 ± 7.2	16.0 ± 5.7	-11.5	-4.4	0.000	13.1 ± 4.9	-8.4	-2.0	0.003
Longitudinal Arch °	160.2 ± 14.3	148.0 ± 9.8	5.8	18.5	0.001	153.1 ± 10.6	-5.6	14.6	0.064
M1 °	14.1 ± 4.1	17.31 ± 4.1	-4.9	-1.5	0.001	16.7 ± 3.9	-1.2	-4.1	0.001
NH (mm)	16.9 ± 7.5	25.7 ± 7.2	-11.1	-6.4	0.000	22.9 ± 6.9	-8.8	-3.1	0.001
CCA °	17.0 ± 10.5	34.3 ± 7.7	-23.0	-11.5	0.000	28.8 ± 7.8	-17.1	-6.1	0.000
MCA °	4.8 ± 3.4	2.9 ± 1.8	0.3	3.7	0.020	2.8 ± 1.9	0.4	3.5	0.013

**Fig. 2:**
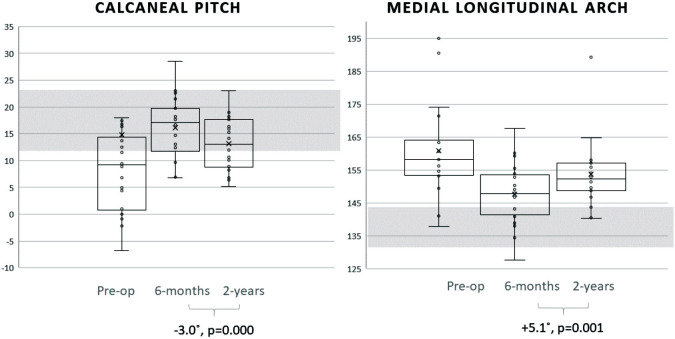
Calcaneal Pitch and Medial Longitudinal Arch measurements pre-surgery, six months and two years post-surgery presented in a box and whiskers plot. Shaded areas represent reference normal radiographical parameters within one standard deviation. At two years post-surgery, none of the angles or heights deteriorated past baseline. All measurements remained significantly corrected for, as compared to pre-surgery.

From the timeframe of 6 months to 2 years post-operatively, there were no significant changes to M1 (p=0.335) or MCA (p=0.862). However, there was a significant increase of the Longitudinal Arch by a mean of 5.1° (P=0.001) (Fig. 2) with a decrease in the Pitch by 2.9° (p=0.003). CCA decreased significantly by 5.5° (p=0.000) with the NH decreasing significantly by 2.8mm (p=0.001).

Osteoarthritis at the naviculocuneiform joint worsened in 12 (54.5%) of feet and arthritis at the medial cuneiform-1st metatarsal joint worsened in 10 (45.4%) over the two years post-surgery. Applying Pearson Chi-Square calculation, the progression in arthritis did not correlate significantly with satisfaction scores (naviculocuneiform joint p=0.427 and medial cuneiform-1st metatarsal joint p=0.089), neither did it correlate with age or BMI (naviculocuneiform joint p=0.809, 0.286 and medial cuneiform-1st metatarsal joint p=0.531, 0.943).

There were significant improvements in VAS and AOFAS hindfoot and midfoot scores at six months post-surgery. All scores improved at the two years follow-up visit. Hindfoot and midfoot VAS scores improved by an average of 4.6 and 3.8 points at 2 years post-surgery (p<0.001). Hindfoot and midfoot AOFAS scores improved by an average of 34.6 and 36.7 points at 2 years post-surgery (p<0.001).

Midfoot AOFAS scores showed statistically significant improvement from 6 months to 2 years post-surgery (p=0.018, CI -20.4 to -2.2) (Table II). In contrast, the improvement in hindfoot AOFAS scores from 6 months to 2 years post-surgery was not statistically significantly (p=0.063, CI -18.9 to 0.5). Using the independent T-tests, there was no statistically significant difference between overall improvement rates for midfoot versus hindfoot scores (VAS p=0.188, AOFAS p=0.976).

**Table II:** Six months post-surgery scores and two years post-surgery scores compared to baseline pre-operative scores, paired T-test.

	Pre-surgery	Six months post-surgery	95% CI of the difference		p-value	Two years post-surgery	95% CI of the difference		p-value
			Lower	Upper			Lower	Upper	
Hind-foot VAS	6.6 ± 2.8	2.6 ± 3.4	2.2	5.9	0.000	2.0 ± 3.3	2.7	6.5	0.000
Hind-foot AOFAS	38.9 ± 19.7	64.4 ± 29.3	-40.6	-10.3	0.002	73.5 ± 25.6	-48.2	-21.1	0.000
Mid-foot VAS	5.1 ± 3.7	1.8 ± 3.3	1.3	5.4	0.003	1.3 ± 2.6	1.8	5.8	0.001
Mid-foot AOFAS	39.1 ± 24.0	65.4 ± 31.1	-42.7	-9.9	0.003	76.7 ± 22.2	-51.5	-23.7	0.000

**Fig. 3:**
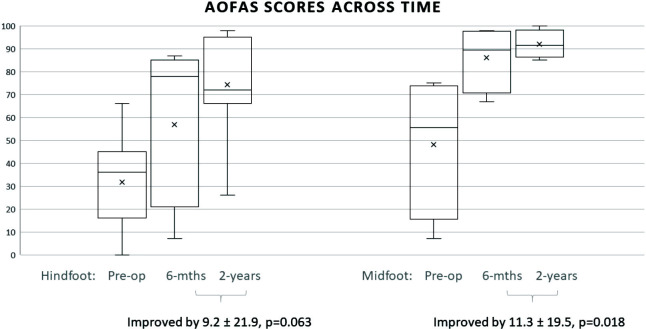
AOFAS scores for hind-foot and mid-foot measured pre-operatively, six months and two years post-operatively.

Two years post-surgery, 19 (86.4%) patients reported being satisfied with the operation and their results. The improvement in hindfoot and midfoot AOFAS scores correlated significantly with patient satisfaction levels at two years post-surgery (Fig. 3). However, satisfaction scores did not show significant correlation with radiological correction (Pearson correlation 0.016, p=0.951).

None of the 22 feet suffered any wound complications post-surgery. There were two (9.1%) feet requiring re-operation, of which one underwent total ankle replacement and the other removal of screws and washout for chronic osteomyelitis. Two (9.1%) feet were offered revision arthrodesis due to Computed Tomography documented non-union of subtalar joints, but the patients decided on conservative management. None of the feet required secondary fusion of the naviculocuneiform or tarsometatarsal joints.

The phone survey revealed a pain score of zero in 15 out of 22 (71.4%) feet, with 16 out of 21 (76.2%) patients being community ambulant without requiring walking aid. Eleven (52.4%) patients reported limitations in their activities and function due to other comorbidities for example previous cerebrovascular accidents or spinal stenosis. Satisfaction was met in 19 out of 22 (86.4%) feet inclusive of the two patients who underwent re-operation.

## Discussion

Our study demonstrated excellent improvements in clinical outcomes with TAO and percutaneous TAL. AOFAS scores improved significantly at six months and continued to improve at two years post-surgery (Fig. 3). There were high rates of patient satisfaction with this procedure, with the majority having no pain and being community ambulant on a phone call survey, at a longer term of 8.3 years.

Radiological measurements six months post-surgery demonstrated correction of the calcaneal pitch and arch. However, subsequent radiographic assessments showed evidence of arch collapse from six months to two years post-surgery. The Medial Longitudinal Arch showed some sagging at the naviculocuneiform joint (Fig. 2). The preserved 1st - Metatarsal declination and Medial Cuneiform-1st Metatarsal Angles suggests a stable medial cuneiform-1st metatarsal joint. Thus, we postulate that most of the stress forces post-operatively occurred in the sagittal plane of the naviculocuneiform joint.

By lengthening the Achilles tendon in our series, in the presence of a solid hind-foot block thorough a triple arthrodesis, the upward vector moment arm on the calcaneum is reduced, therefore contributing to a reduction in the forces on the medial column. In our series, radiological angles and heights were maintained at two years post-surgery. In contrast, Meary was unable to correct, nor maintain the angles at final follow-up in his Triple Arthrodesis series^10^. The deformation generated by the naviculocuneiform joint was less pronounced in our series compared to Meary’s series. We postulate that the naviculocuneiform sag, seen radiographically at two years, may be an early sign of consequent arch collapse, which probably was slowed by the additional TAL. TA lengthening decreases forefoot contact pressures^27-29^. It has been shown to reduce stressors on the forefoot and midfoot, with peak loading pressures being transferred to the hindfoot^28-31^. Failure to identify and address a tight posterior compartment during triple arthrodesis can lead to an exacerbating force in hindfoot deformity and excessive midfoot stresses^16^. Our significantly improved midfoot AOFAS scores are the result of decrease in forefoot and midfoot loading from Achilles tendon lengthening.

Our study is the first to report solely on Tendo-Achilles lengthening in triple arthrodesis surgery for AAFD, reporting radiographic angles and clinical outcomes at different time frames, which document the progression of disease. In the last 20 years, literature on the results of triple arthrodesis in AAFD has been sparse. There has been a focus on extended fusions of the first tarsometatarsal joints^30^ and medial column fusions^31^. However, this translates to longer operative times and a more extensive surgery. In contrast, Achilles tendon lengthening is a simple procedure which is now commonly done percutaneously to minimise surgical site complications. TAL reportedly incurs temporary weakening of the tendon which reduces the plantarflexion moment on the calcaneus^28^. This fortuitously decreases the pressures on the arch while the patient is in the early post-operative phase. None of the patients in our series developed complications particular to the Achilles tendon lengthening.

Our study gives us clinical insight to how radiographic angles may not correlate with clinical scores and patient satisfaction scores. Thus, it is important to assess the patient clinically and take efforts to address the various components of the AOFAS score to improve the patient’s outcome.

Limitations of this study are our inability to compare this group of patients to a control Triple arthrodesis group without Tendo-Achilles tendon lengthening. Secondly, 11 (52.4%) of our study patients had comorbidities that limited their walking during the telephone survey. Therefore, it was challenging to discern to what extent triple arthrodesis with TAL contributed to their final functional limitations.

## Conclusion

Triple Arthrodesis with concomitant tendoachilles lengthening is associated with good clinical and radiographic outcomes as well as high satisfaction rates. Despite evidence of mid-foot sagging at two years mark, there was a continued improvement in clinical outcome scores. Longer term follow-up with clinical scores and imaging for patients may provide further insight into this phenomenon.
